# Physical literacy and mental well-being in adults: a cross-sectional examination of their association and links with physical activity engagement

**DOI:** 10.3389/fpsyg.2026.1908692

**Published:** 2026-07-17

**Authors:** Bekir Erhan Orhan, Walaa Jumah Alkasasbeh, Aydın Karaçam, Luís Branquinho, Adam Tawfiq Amawi

**Affiliations:** 1 Faculty of Sports Sciences, Istanbul Aydın University, Istanbul, Türkiye; 2 Department of Physical Education, Faculty of Sport Science, The University of Jordan, Amman, Jordan; 3 Faculty of Sport Sciences, Bandırma Onyedi Eylül University, Balıkesir, Türkiye; 4 Biosciences School of Elvas, Polytechnic University of Portalegre, Elvas, Portugal; 5 Life Quality Research Center (LQRC-CIEQV), Santarem, Portugal; 6 Research Center in Sports Sciences, Health Sciences and Human Development, Covilhã, Portugal; 7 Centro De Investigação Do Instituto Superior De Ciência Educativas (CI-ISCE), Penafiel, Portugal; 8 Department of Movement Sciences and Sports Training, School of Sports Science, The University of Jordan, Amman, Jordan

**Keywords:** health promotion, mental well-being, physical activity, physical literacy, self-determination theory, sustainable health

## Abstract

**Background:**

Physical literacy, the integrated motivation, confidence, physical competence, knowledge, and understanding that underpins lifelong physical activity, has been advanced as a holistic determinant of health. Yet, evidence linking it to positively framed mental well-being in adults remains scarce and is dominated by samples of children and adolescents.

**Aim:**

This study examined the association between physical literacy and mental well-being in Turkish adults and tested whether both constructs differed by sex, educational attainment, athletic background, regular exercise participation, and habitual physical activity level.

**Methods:**

In a cross-sectional design, 375 adults (64.5% male; mean age = 32.23 years, SD = 11.03, range 18–71), a convenience sample skewed toward physically active and tertiary-educated men, completed validated Turkish measures of physical literacy and mental well-being. Active-living exposures were assessed using a single self-report item. Internal consistency was high (physical literacy α = 0.89, ω = 0.90; mental well-being α = 0.92, ω = 0.92). Pearson correlations, *t*-tests, one-way ANOVAs, and hierarchical regression were computed with effect sizes and 95% confidence intervals; discriminant validity was tested directly, and an exploratory mediation was examined.

**Results:**

Physical literacy was moderately and positively associated with mental well-being (*r* = 0.45, *p* < 0.001), and the two constructs showed evidence of empirical distinctness (HTMT = 0.52, 95% CI [0.42, 0.63]). Adults with an athletic background and regular exercisers reported higher physical literacy (*d* = 0.83 and 1.09) and mental well-being (*d* = 0.36 and 0.63), and both outcomes increased in an ordered gradient across habitual activity-frequency categories (physical literacy η^2^ = 0.21; mental well-being η^2^ = 0.07). Physical literacy explained 11% of incremental variance in well-being beyond demographic and behavioral covariates (β = 0.41). Men scored marginally higher than women on both measures, educational attainment was weakly related to mental well-being but not physical literacy, and physical literacy declined modestly with age (*r* = −0.18).

**Conclusion:**

Higher physical literacy co-occurs with better mental well-being in adults and remains associated with mental well-being after adjustment for covariates, positioning it as a candidate target for adult mental health promotion. The cross-sectional design and active, educated convenience sample preclude causal and population-level inference, and independent confirmatory validation of the adapted instrument is needed before these findings are treated as more than provisional.

## 1 Introduction

Physical literacy has emerged over the past two decades as an organizing concept for understanding why some individuals sustain active lifestyles across the life course while others do not (Whitehead, 2001; Cairney et al., 2019; Young et al., 2020). Rather than reducing movement to fitness or motor performance, the construct is multidimensional, integrating an individual’s motivation, confidence, physical competence, and the knowledge and understanding required to value and take responsibility for lifelong engagement in physical activity (Edwards et al., 2017; International Physical Literacy Association., 2017 Carl et al., 2022; Cairney et al., 2019). This holistic framing positions physical literacy not merely as a correlate of physical health but as a psychosocial resource with plausible implications for mental health and well-being (Britton et al., 2023; Ma et al., 2021; Tang et al., 2023).

The affective and cognitive components of physical literacy, namely self-confidence in movement contexts, intrinsic motivation, and an understanding of how and why to be active, overlap conceptually with established psychological determinants of well-being (Tang et al., 2023; Jefferies et al., 2019). Mental well-being, in the eudaimonic and hedonic tradition, encompasses positive affect, satisfying interpersonal relationships, and positive functioning such as autonomy and competence (Keyes, 2002; Tennant et al., 2007; Ruggeri et al., 2020). To the extent that physically literate individuals experience greater perceived competence, autonomy, and social connectedness through movement, the basic psychological needs whose satisfaction underpins eudaimonic well-being within self-determination theory (Ryan and Deci, 2000; Teixeira et al., 2012; Ntoumanis et al., 2021), physical literacy may foster the psychological assets that constitute mental well-being. Self-determination theory (Ryan et al., 2022; Vasconcellos et al., 2020) is invoked here as an interpretive framework for why the two constructs might co-occur rather than as a model this study sets out to test, since the present design includes no measure of basic-need satisfaction; the proposed psychological pathway is therefore advanced as a hypothesis for future work. Consistent with this reasoning, physical activity produces medium-sized reductions in depressive, anxious, and distress symptoms across adult populations and is further associated with higher positively framed subjective well-being (Singh et al., 2023; Buecker et al., 2021). Physical literacy is itself a robust correlate of physical-activity participation (Naylor et al., 2025), and the motivation and enjoyment that sustain movement are established determinants of continued engagement (Orhan et al., 2025a).

Empirical work directly linking physical literacy to mental health, however, has concentrated almost exclusively on young people (Ma et al., 2021; Britton et al., 2023). In a large sample of college students, physical literacy was positively related to mental health, and this association was mediated by resilience (Ma et al., 2021). A parallel study of college students linked physical literacy to positive self-esteem, a psychological resource closely tied to well-being (She et al., 2023). Among children and adolescents, physical literacy has been associated with higher positive affect, vitality, and overall well-being and with lower negative affect (Britton et al., 2023; Melby et al., 2022, 2023), and the resilience-mediated pathway has since been reproduced in a moderated-mediation model among adolescents (Ma et al., 2021; Zhu et al., 2025). Direct evidence in adults remains sparse: a recent cross-sectional study found that adults reporting a mental-health diagnosis scored lower on physical literacy than those without one (Naylor et al., 2026), establishing a group-level deficit but leaving open whether physical literacy varies continuously with positively framed mental well-being across the general adult range. The adult population, by contrast, remains markedly underrepresented in the physical-literacy literature, despite adults facing distinct barriers to activity, including time constraints, occupational demands, and reduced leisure opportunity, such as the displacement of leisure-time movement by screen-based and sedentary technology use (Orhan et al., 2025b), and bearing a disproportionate share of the global burden of physical inactivity and its health sequelae (World Health Organization, 2022; Guthold et al., 2018).

Beyond the central association, several demographic and behavioral factors warrant attention, as they may structure both constructs. Sex differences in physical literacy and well-being are reported inconsistently and warrant examination in a Turkish adult sample (Albéniz-Pérez et al., 2025; Mudrinic et al., 2025). Educational attainment is a candidate correlate of mental well-being through its links to socioeconomic resources and health literacy. Most directly, an athletic background, regular exercise participation, and habitual physical activity level index cumulative exposure to movement and should, if physical literacy is meaningfully tied to active living, differentiate individuals on both physical literacy and well-being. Establishing these known-groups differences also provides convergent evidence for the construct validity of the adapted instrument in everyday use.

Accordingly, the present study pursued three aims. First, to quantify the association between physical literacy and mental well-being in a sample of Turkish adults. Second, to determine whether physical literacy and mental well-being differ by sex, educational attainment, athletic background, regular exercise participation, and habitual physical activity level. Third, to test whether physical literacy accounts for variance in mental well-being beyond demographic and behavioral correlates and whether it helps explain the link between active living and well-being. We hypothesized that (H1) physical literacy would be positively associated with mental well-being; (H2) adults with an athletic background, those exercising regularly, and those with higher habitual activity levels would report higher physical literacy and higher mental well-being; (H3) a graded gradient would be observed across activity-frequency categories for both outcomes; and (H4) physical literacy would explain incremental variance in mental well-being after demographic and behavioral covariates were controlled. Given that physical literacy is conceptually proximal to active living, in that its motivational and confidence facets overlap with the self-perceptions that accompany regular activity, we examined, only on an exploratory and hypothesis-generating basis, whether physical literacy statistically accounts for the association between habitual activity level and mental well-being, and which of its five subdimensions carries the association. Because the predictor and the candidate mediator overlap conceptually and the design is cross-sectional, this analysis is not interpreted as evidence of a causal pathway. Sex and educational differences were examined without directional hypotheses.

## 2 Materials and methods

### 2.1 Design and participants

A cross-sectional, correlational survey design was used. A convenience sample of 375 adults residing in Türkiye completed an online questionnaire. Participants were recruited through online convenience and snowball sampling: an invitation containing the study information sheet and the survey link was circulated through institutional and social-media networks, and recipients were invited to forward it to other eligible adults. To be eligible, respondents had to be 18 years of age or older, currently residing in Türkiye, able to read Turkish, and willing to provide informed consent; responses without completed consent were not retained, and no further exclusion criteria were applied. Before analysis, the dataset was screened for out-of-range values, incomplete records, and duplicated or internally inconsistent response patterns, none of which were identified. Because recruitment relied on an open link rather than a closed panel, and because no attention-check or instructed-response items were embedded, the scope for detecting careless or duplicate responding was limited, a constraint revisited in Section “4.1 Limitations and future directions.” Participants ranged in age from 18 to 71 years (M = 32.23, SD = 11.03) and were predominantly male (*n* = 242, 64.5%; female *n* = 133, 35.5%). With respect to educational attainment, 120 participants (32.0%) had completed secondary (high-school) education, 174 (46.4%) held an undergraduate degree, and 81 (21.6%) held a postgraduate degree. Most participants reported an athletic background (*n* = 291, 77.6%) and engagement in regular exercise (*n* = 242, 64.5%). Habitual physical activity was distributed across four levels: no activity (*n* = 30, 8.0%), low (1–2 days/week; *n* = 145, 38.7%), moderate (3–4 days/week; *n* = 143, 38.1%), and high (≥ 5 days/week; *n* = 57, 15.2%). Participation was voluntary and anonymous, and informed consent was obtained before data collection. The study protocol was approved by the Social and Human Sciences Ethics Committee of Istanbul Aydın University (meeting no. 2025/10; 18 September 2025) and was conducted in accordance with the Declaration of Helsinki. The study is reported in line with the STROBE guideline for cross-sectional studies.

Because the sample size was fixed before analysis, a sensitivity power analysis was conducted in G*Power 3.1.9.7 (Faul et al., 2009) to determine the smallest effects detectable at the achieved sample size, with α = 0.05 (two-tailed) and power = 0.80. For the bivariate correlations (H1), the design was sensitive to *r* ≥ 0.14 (*N* = 375). For the focal incremental test in the hierarchical regression (H4), it was sensitive to a single-predictor effect of f^2^ ≥ 0.02 (df1 = 1, df^2^ = 366), corresponding to an R^2^ increment of approximately 0.02. For the independent-samples *t*-tests (H2), the minimum detectable standardized difference ranged from *d* = 0.30 for the more balanced contrasts (sex; *n* = 133 vs. 242) to *d* = 0.35 for the most unbalanced (athletic background; *n* = 84 vs. 291). For the one-way ANOVAs, the design was sensitive to *f* = 0.16 (η^2^ = 0.025) across the three education groups and *f* = 0.17 (η^2^ = 0.029) across the four activity-frequency groups. The sample was also adequate to detect indirect effects arising from small-to-medium constituent paths (Fritz and MacKinnon, 2007). All focal effects observed in the present study (*r* = 0.45; regression *f*^2^ = 0.14; activity-level η^2^ = 0.07–0.21) exceeded these minimum detectable thresholds, indicating that the principal analyses were adequately powered. The smallest subgroup (no-activity, *n* = 30) was, however, underpowered for small effects, so the pairwise contrasts involving that group are interpreted with caution (Section “3.4 Group differences: education and habitual activity level”). The sample provided a participant-to-item ratio above 15:1 for the psychometric checks reported below. The realized sample was predominantly male, physically active, and tertiary-educated and is therefore best characterized as a convenience sample of engaged adults rather than a representative cross-section of the Turkish adult population; this composition is revisited when generalizability is considered (Section “4.1 Limitations and future directions”).

### 2.2 Measures

#### 2.2.1 Physical literacy in adults scale (PLAS)

Physical literacy was assessed with the Turkish adaptation (Orhan and Karaçam, 2025) of the 23-item PLAS (Naylor et al., 2024). The Turkish version comprises five factors (Motivation, Social, Confidence, Physical [coordination and strength], and Knowledge) and yields a single total score, with one item (“I am not physically strong”) reverse-scored; higher scores indicate greater physical literacy. Response formats are heterogeneous, inherited from the source items: Motivation (5 items) and Social (4 items) rated 1–7, Physical (6 items) 1–8, Knowledge (3 items) 1–5, and Confidence (5 items) rated 1–10 for three items and 1–5 for the remaining two, so that summing the 23 items yields a possible range of 23–166. Whereas the original scale spans four conceptual domains (physical, psychological, social, and cognitive) and its 23 items resolved empirically into six factors (motivation, confidence, social, coordination, strength, and knowledge; Naylor et al., 2024), the Turkish adaptation resolved into five factors, the coordination and strength items loading as a single Physical factor; the implications of this divergence for cross-cultural comparability are addressed in the Discussion. The original Turkish validation reported acceptable model fit, 64.64% explained variance, and α = 0.88 (Orhan and Karaçam, 2025). Because the present analyses used the summed-item total rather than the developer-specified mean composite, they were re-estimated with the mean composite to confirm that the scoring choice did not drive the findings (Supplementary material). In the present sample, internal consistency was high for the total score (Cronbach’s α = 0.89, 95% CI [0.87, 0.90]; McDonald’s ω = 0.90, 95% CI [0.88, 0.91]) and acceptable-to-good for the subscales (α = 0.76–0.88). Total scores approximated normality (skewness = −0.46, kurtosis = 0.45).

#### 2.2.2 Warwick-edinburgh mental well-being scale (WEMWBS)

Mental well-being was measured with the Turkish form (Keldal, 2015) of the 14-item WEMWBS (Tennant et al., 2007). Items are rated on a 5-point scale (1 = strongly disagree to 5 = strongly agree), and all items are positively worded, yielding total scores from 14 to 70, with higher scores reflecting greater mental well-being. The Turkish form is unidimensional and was originally reported to have a Cronbach’s α of 0.92. In the present sample, internal consistency was likewise high (Cronbach’s α = 0.92, 95% CI [0.91, 0.93]; McDonald’s ω = 0.92, 95% CI [0.91, 0.94]). Total scores were moderately negatively skewed but within conventionally acceptable limits (skewness = –0.94, kurtosis = 1.00).

#### 2.2.3 Active-living exposures

Athletic background (yes/no), regular exercise participation (yes/no), and habitual physical activity level (no activity; 1–2; 3–4; ≥ 5 days/week) were each assessed with a single self-report item. The three items, presented in Turkish, were worded as follows. Athletic background was assessed with “Do you have a background as an athlete (for example, licensed, club, or competitive sport participation)?” (response options: yes/no). Regular exercise participation was assessed with “Do you currently engage in regular exercise?” (yes/no). Habitual physical activity level was assessed with “On how many days in a typical week do you engage in physical activity?,” with four ordered response categories: none, 1–2 days, 3–4 days, and 5 or more days per week. These items index frequency of, and self-reported exposure to, active living rather than activity intensity, duration, or total volume; this scope is addressed in Section “4.1 Limitations and future directions.”

### 2.3 Statistical analysis

Analyses were conducted in IBM SPSS Statistics (v.25) with the PROCESS macro (v4.2; Hayes, 2022); McDonald’s ω with bootstrap confidence intervals (Dunn et al., 2014), the discriminant-validity models, and the supplementary one-factor solutions were estimated in R (v4.3). Before inferential testing, distributions were screened for normality (skewness and kurtosis within ± 2 and ± 7, respectively) and homogeneity of variance (Levene’s test). To confirm that the five-factor PLAS structure held in the present sample, the five-factor structure identified by Orhan and Karaçam (2025) was tested with a confirmatory factor analysis on the present data (R, lavaan), with fit indices reported in Section “3.1 Descriptive statistics.” Because physical literacy and mental well-being are conceptually proximal, their discriminant validity was tested directly by comparing a two-factor measurement model (separating the constructs) with a one-factor model collapsing them, and by computing the heterotrait-monotrait ratio of correlations (HTMT; Henseler et al., 2015). The physical-literacy-well-being association was estimated with Pearson’s r; associations with age were examined in the same correlation matrix. Sex, athletic background, and regular-exercise differences were tested with independent-samples *t*-tests, with Welch’s correction applied where Levene’s test was significant; Cohen’s d (with Hedges’ correction) quantified effect size. Differences across educational attainment and habitual activity level were tested with one-way ANOVA, with η^2^ as the effect-size index and least-significant-difference (LSD) pairwise comparisons, with Tukey HSD and Games-Howell comparisons additionally examined as familywise-error checks; where the homogeneity-of-variance assumption was violated, Welch’s F and Games-Howell comparisons were computed and are reported alongside the standard tests in Section “3.4 Group differences: education and habitual activity level.” Where Levene’s test indicated heterogeneity of variance, the Welch-corrected omnibus test and the Games-Howell pairwise procedure were treated as the primary basis for inference, and the homoscedastic LSD results are retained only for completeness.

To establish the incremental contribution of physical literacy beyond demographic and behavioral covariates, a three-step hierarchical multiple regression predicted mental well-being from (Step 1) age, sex, and education; (Step 2) athletic background, regular exercise, and habitual activity level; and (Step 3) total physical literacy, with the change in R^2^ and f^2^ evaluated at each step. Regression assumptions were verified using tolerance and variance inflation factors (VIFs), the Durbin-Watson statistic, and inspection of standardized residual plots. Because the bivariate and known-groups analyses suggested that physical literacy might transmit the activity-well-being association, a simple mediation model (PROCESS Model 4) tested, on an exploratory basis, the indirect effect of habitual activity level on mental well-being through physical literacy, adjusting for age and sex; a complementary moderation model (PROCESS Model 1) tested whether the physical-literacy-well-being slope varied across activity levels. The four-level activity variable was entered as continuous in these models; its coding as an ordered-categorical predictor was examined as a sensitivity check. Indirect and conditional effects were estimated with 5,000 percentile bootstrap resamples (seed = 31216; Preacher and Hayes, 2008), with inference based on 95% percentile bootstrap confidence intervals that exclude zero. Finally, the five PLAS subdimensions were entered simultaneously as predictors of mental well-being to identify which facets carried the association, with the family of zero-order subscale correlations adjusted using the Benjamini-Hochberg false-discovery-rate (FDR) procedure. Because all data were self-reported from a single source, common-method bias was assessed with Harman’s single-factor test. Hypotheses H1–H4 were treated as confirmatory, whereas the mediation, moderation, and subdimension analyses were exploratory; the study was not preregistered. All tests were two-tailed with α set at 0.05, and 95% confidence intervals are reported for mean differences, effect sizes, regression coefficients, and indirect effects. Missing data were negligible (confined to two cells on a single Social-subscale item; ≤ 0.6% on any item) and handled by pairwise deletion.

## 3 Results

### 3.1 Descriptive statistics

On average, participants reported moderately high physical literacy (M = 114.65, SD = 19.82; range 30–165) and mental well-being (M = 57.34, SD = 10.00; range 14–70). A confirmatory factor analysis re-estimating the five-factor PLAS structure on the present sample (*n* = 373; the two participants with missing data on one PLAS item were excluded listwise, so this model alone draws on 373 of the full sample of 375; maximum-likelihood estimation) produced fit that was acceptable on residual-based but marginal on incremental indices: χ^2^(220) = 829.70, *p* < 0.001, χ^2^/df = 3.77, CFI = 0.86, TLI = 0.84, RMSEA = 0.086 (90% CI [0.080, 0.093]), SRMR = 0.078. Fit was therefore weaker than in the original Turkish validation (which reported CFI = 0.90, RMSEA = 0.06), with CFI and TLI just below and RMSEA marginally above conventional thresholds; the five-factor structure was retained for comparability with the source instrument, and the implications of this modest fit are considered in Section “4.1 Limitations and future directions.” Descriptive statistics for the total sample and by grouping variable are presented in Table 1.

**TABLE 1:** Descriptive statistics for physical literacy and mental well-being by participant characteristics.

Variable / group	*n*	Physical literacy M (SD)	Mental well-being M (SD)
Total sample	375	114.65 (19.82)	57.34 (10.00)
Women	133	110.95 (20.01)	55.88 (11.38)
Men	242	116.69 (19.47)	58.14 (9.07)
Education: high school	120	115.38 (18.38)	56.20 (9.78)
Education: undergraduate	174	114.25 (20.90)	57.01 (10.62)
Education: postgraduate	81	114.43 (19.73)	59.74 (8.54)
Athletic background: yes	291	118.14 (17.77)	58.14 (9.23)
Athletic background: no	84	102.56 (21.85)	54.57 (11.93)
Regular exercise: yes	242	121.43 (16.11)	59.46 (8.28)
Regular exercise: no	133	102.32 (20.05)	53.47 (11.61)
Activity: none	30	95.73 (24.22)	49.83 (15.32)
Activity: low (1–2 d/wk)	145	108.11 (19.89)	56.44 (10.26)
Activity: moderate (3–4 d/wk)	143	120.04 (14.60)	58.72 (7.81)
Activity: high ( ≥ 5 d/wk)	57	127.72 (14.57)	60.11 (8.68)

### 3.2 Association between physical literacy and mental well-being

Supporting H1, physical literacy was moderately and positively correlated with mental well-being, r(373) = 0.45, *p* < 0.001, indicating that adults with higher physical literacy tended to report greater mental well-being; the shared variance was approximately 20% (r^2^ = 0.20). Age was weakly and negatively associated with physical literacy, r(373) = −0.18, *p* < 0.001, but was unrelated to mental well-being, r(373) = 0.07, *p* = 0.160 (Table 2). The same association emerged when physical literacy was scored as the developer-specified mean-based composite rather than the summed-item total (Supplementary material), indicating that the finding is not an artifact of the scoring metric.

**TABLE 2:** Pearson correlations among age, physical literacy, and mental well-being (*N* = 375).

Variable	1	2	3
1. Age	–		
2. Physical literacy	−0.18***	–	
3. Mental well-being	0.07	0.45***	–

***p < 0.001. Dashes denote the diagonal.

Because the two constructs are conceptually proximal, their empirical distinctness was tested directly. A model specifying physical literacy and mental well-being as two correlated latent factors fit substantially better than a one-factor model collapsing them, Δχ^2^(1) = 1241.3, *p* < 0.001, ΔCFI = 0.16, and the heterotrait-monotrait ratio of correlations was well below the conservative 0.85 threshold, HTMT = 0.52, 95% bootstrap CI [0.42, 0.63], with the interval excluding 1.0 (Henseler et al., 2015); the estimated latent correlation between the constructs was 0.52. That the HTMT and the latent factor correlation converged on the same value is unsurprising, as both index the disattenuated (measurement-error-corrected) association between the constructs. These convergent results indicate that physical literacy and mental well-being are empirically distinguishable despite their moderate association. Because physical literacy is multidimensional, the single-PL-factor measurement models showed limited absolute fit; the HTMT criterion, which is robust to this, is therefore the primary basis for the discriminant-validity conclusion. The HTMT was computed treating the physical-literacy items as indicators of a single construct; the resulting estimate should therefore be read as an approximation pending a higher-order model that represents the five-factor structure. This result bears directly on the interpretation of the incremental-validity (Section “3.5 Incremental validity of physical literacy”) and subdimension (Section “3.7 Which subdimensions carry the association”) analyses.

### 3.3 Group differences: sex, athletic background, and regular exercise

Independent-samples *t*-tests revealed small but significant sex differences: men scored higher than women on both physical literacy, t(373) = −2.70, *p* = 0.007, *d* = −0.29, 95% CI [−0.50, −0.08], and mental well-being, t(373) = −2.11, *p* = 0.036, *d* = −0.23, 95% CI [−0.44, −0.02]; for the latter, Levene’s test was borderline (*p* = 0.05) and the Welch-corrected result remained at the conventional significance threshold, so this difference should be interpreted with caution. Consistent with H2, adults with an athletic background reported substantially higher physical literacy, t(373) = 6.71, *p* < 0.001, *d* = 0.83, 95% CI [0.58, 1.08] (Welch t(116.5) = 5.99), and higher mental well-being, t(373) = 2.91, *p* = 0.004, d = 0.36, 95% CI [0.12, 0.60] (Welch t(113.2) = 2.53, *p* = 0.013). The largest differences were associated with regular exercise: regular exercisers reported markedly higher physical literacy, t(373) = 10.05, *p* < 0.001, *d* = 1.09, 95% CI [0.86, 1.31], and higher mental well-being, t(373) = 5.79, *p* < 0.001, *d* = 0.63, 95% CI [0.41, 0.84] (Welch t(207.3) = 5.26, *p* < 0.001). Table 3 summarizes these comparisons.

**TABLE 3:** Independent-samples *t*-tests for physical literacy and mental well-being by sex, athletic background, and regular exercise.

Outcome / contrast	Group M (SD)	t	*p*	d	95% CI for d
PL - Sex	W 110.95 / M 116.69	−2.70	0.007	−0.29	[−0.50, −0.08]
WB - Sex	W 55.88 / M 58.14	−2.11	0.036	−0.23	[−0.44, −0.02]
PL - Athletic bkgd.	Yes 118.14 / No 102.56	6.71	< 0.001	0.83	[0.58, 1.08]
WB - Athletic bkgd.	Yes 58.14 / No 54.57	2.91	0.004	0.36	[0.12, 0.60]
PL - Regular exercise	Yes 121.43 / No 102.32	10.05	< 0.001	1.09	[0.86, 1.31]
WB - Regular exercise	Yes 59.46 / No 53.47	5.79	< 0.001	0.63	[0.41, 0.84]

PL, physical literacy; WB, mental well-being; W, women; M, men; d, Cohen’s d (Hedges’-corrected estimates were near-identical). Negative d indicates women < men. Welch’s correction was applied where Levene’s test was significant (athletic background and regular exercise).

### 3.4 Group differences: education and habitual activity level

Educational attainment was unrelated to physical literacy, *F*(2, 372) = 0.12, *p* = 0.887, η^2^ = 0.001, but was weakly associated with mental well-being, *F*(2, 372) = 3.25, *p* = 0.040, η^2^ = 0.017. LSD comparisons indicated that postgraduate-educated adults (M = 59.74) reported higher well-being than those with high-school (M = 56.20; *p* = 0.014) or undergraduate (M = 57.01; *p* = 0.041) education, though the effect was small. Under familywise-corrected comparisons, however, only the postgraduate-high-school difference remained reliable (Tukey HSD *p* = 0.036; Games-Howell *p* = 0.020), whereas the postgraduate-undergraduate difference did not (*p* = 0.103 and 0.074, respectively); this small education effect is therefore carried by the high-school-postgraduate contrast and should be interpreted with caution.

Habitual physical activity level produced the strongest and most consistent gradient, supporting H2 and H3; this gradient is interpreted as a graded, ordered association across activity-frequency categories rather than a dose-response relationship, because the exposure indexed frequency (days/week) only and not intensity, duration, or volume (Section “4.1 Limitations and future directions”). Physical literacy differed substantially across the four activity-frequency categories, *F*(3, 371) = 32.83, *p* < 0.001, η^2^ = 0.21 (a large effect), increasing in order from no activity (M = 95.73) through low (M = 108.11) and moderate (M = 120.04) to high activity (M = 127.72); all six pairwise comparisons were significant under LSD (*p* ≤ 0.006). Mental well-being showed the same ordering, *F*(3, 371) = 8.93, *p* < 0.001, η^2^ = 0.07 (a medium effect), rising from no activity (M = 49.83) to high activity (M = 60.11); under LSD, pairwise differences were significant except between the moderate and high groups (*p* = 0.362). Because Levene’s test was significant for both outcomes in this analysis, Welch’s F and Games-Howell comparisons are the appropriate robust confirmation. The Welch-corrected omnibus tests confirmed both effects (physical literacy: Welch’s *F*(3, 102.20) = 29.26, *p* < 0.001; mental well-being: Welch’s *F*(3, 99.64) = 5.42, *p* = 0.002). Under the more conservative Games-Howell procedure, two contrasts that were significant under LSD did not survive: for physical literacy the no-activity vs. low-activity difference was no longer significant, and for mental well-being only the no-activity group differed reliably from the moderate and high groups (all remaining well-being contrasts non-significant). The omnibus effects and the overall ordered gradient across activity-frequency categories are therefore robust, although the small no-activity group (*n* = 30) drives the affected pairwise contrasts. Table 4 reports the ANOVA results.

**TABLE 4:** One-way ANOVAs for physical literacy and mental well-being by education and habitual physical activity level.

Outcome / factor	F (df)	*p*	η^2^	Levene p	Pattern
PL - Education	0.12 (2,372)	0.887	0.001	0.122	ns
WB - Education	3.25 (2,372)	0.040	0.017	0.193	Postgrad > HS, UG
PL - Activity level	32.83 (3,371)	< 0.001	0.21	0.020	None < Low < Mod < High
WB - Activity level	8.93 (3,371)	< 0.001	0.07	< 0.001	None < Low < Mod ≈ High

PL, physical literacy; WB, mental well-being; HS, high school; UG, undergraduate; ns, non-significant. For the activity-level analyses, Levene’s test was significant; Welch’s F and Games-Howell comparisons are recommended for confirmation.

### 3.5 Incremental validity of physical literacy

A three-step hierarchical regression tested whether physical literacy predicted mental well-being, controlling for demographic and behavioral covariates (Table 5). Demographic variables (age, sex, education) accounted for a small share of variance, R^2^ = 0.03, *F*(4, 370) = 2.62, *p* = 0.035. Adding the behavioral indicators of active living produced a significant increment, ΔR^2^ = 0.10, ΔF(3, 367) = 14.69, *p* < 0.001, f^2^ = 0.12. Critically. Supporting H4, physical literacy explained a further 11% of variance over and above all covariates, ΔR^2^ = 0.11, ΔF(1, 366) = 52.83, *p* < 0.001, f^2^ = 0.14. It was by a wide margin the strongest predictor in the final model, β = 0.41, 95% CI [0.30, 0.52], *p* < 0.001. Once physical literacy was included, regular exercise retained a small but statistically significant unique coefficient (β = 0.13, *p* = 0.028). In contrast, athletic background and habitual activity level were no longer uniquely predictive (both *p* > 0.15). This indicates that the bivariate associations of athletic background and activity volume with well-being operated largely through physical literacy. In contrast, a small residual association of regular exercise persisted in the adjusted model. Age emerged as a small positive predictor in the adjusted model, β = 0.13, *p* = 0.022, a suppression pattern relative to its null zero-order correlation. This pattern arises because physical literacy is negatively associated with age (*r* = −0.18): once physical literacy is held constant, the positive contribution of age to well-being that it had masked becomes detectable. The model explained 24% of the variance in mental well-being (R^2^ = 0.24; adjusted R^2^ = 0.22; *F*(8, 366) = 14.55, *p* < 0.001). Assumptions were satisfied: all VIFs ≤ 1.89 (tolerances ≥ 0.53) and the Durbin-Watson statistic was 1.98. Because physical literacy and mental well-being share a self-report method and overlap conceptually (Section “3.2 Association between physical literacy and mental well-being”), this 11% increment is best read as an upper bound on the unique contribution of physical literacy: part of it may reflect shared method and construct variance rather than a wholly distinct predictor.

**TABLE 5:** Hierarchical regression predicting mental well-being (*N* = 375).

Step / predictor	β (final)	95% CI	R^2^	Δ R^2^	Δ F (df)	p (Δ F)
Step 1: Demographics			0.03	0.03	2.62 (4, 370)	0.035
Step 2: + Active living			0.13	0.10	14.69 (3, 367)	< 0.001
Step 3: + Physical literacy			0.24	0.11	52.83 (1, 366)	< 0.001
Age	0.13	[0.02, 0.25]				0.022
Sex (men vs. women)	0.03	[−0.07, 0.12]				0.563
Regular exercise	0.13	[0.01, 0.25]				0.028
Habitual activity level	−0.01	[−0.13, 0.11]				0.850
Physical literacy	0.41	[0.030, 0.52]				< 0.001
Education (undergraduate vs. high school)	−0.02	[−0.13, 0.10]				0.800
Education (postgraduate vs. high school)	0.08	[−0.04, 0.21]				0.178
Athletic background (yes vs. no)	−0.01	[−0.11, 0.09]				0.891

β, standardized regression coefficient from the final model; f^2^ = 0.12 (Step 2) and 0.14 (Step 3). Education dummies and athletic background were now reported in full in the rows above; each was non-significant in the final model (all *p* > 0.15). Sex coded 0 = women, 1 = men.

### 3.6 Exploratory analysis: physical literacy and the activity-well-being association

Because physical literacy attenuated the unique contributions of athletic background and habitual activity level in the regression, an exploratory model (PROCESS Model 4; 5,000 bootstrap resamples; seed = 31216) examined whether physical literacy statistically accounts for the association between habitual activity level and mental well-being, adjusting for age and sex. Habitual activity level was associated with physical literacy (*a* = 11.81, *p* < 0.001), and physical literacy with well-being controlling for activity level (*b* = 0.20, *p* < 0.001); the indirect path was distinguishable from zero, ab = 2.41, 95% percentile bootstrap CI [1.60, 3.35]. This pattern is reported solely as hypothesis-generating and is not interpreted as mediation, for three reasons that operate jointly: the cross-sectional design cannot establish temporal precedence; the candidate mediator (physical literacy, which incorporates motivation and confidence for activity) overlaps conceptually with the predictor (activity frequency), so the indirect path may partly reflect construct redundancy rather than transmission; and the predictor is a single ordinal item. For the same reasons, the proportion-mediated index is not reported. A complementary moderation model (PROCESS Model 1) indicated that the physical-literacy-well-being association did not vary across activity levels (interaction *b* = 0.001, *t* = 0.05, *p* = 0.963), so the association was comparable regardless of activity frequency. A definitive test of the proposed ordering requires longitudinal, latent-variable mediation with a validated activity measure (Section “4.1 Limitations and future directions”).

### 3.7 Which subdimensions carry the association

To identify the facets of physical literacy most relevant to well-being, the five PLAS subdimensions were correlated with mental well-being and then entered simultaneously as predictors of mental well-being (Table 6). All five subscales correlated positively with well-being (FDR-corrected *q* < 0.001), with the Physical (coordination and strength) subscale showing the strongest zero-order association (*r* = 0.45). In the simultaneous model, which explained 27% of the variance (R^2^ = 0.27), only the Physical (β = 0.40, *p* < 0.001) and Social (β = 0.24, *p* < 0.001) subscales remained uniquely predictive; the Motivation, Knowledge, and Confidence subscales did not contribute independent variance once the others were controlled. The well-being association therefore appears to be driven primarily by perceived physical competence and the social dimension of physical literacy, rather than by motivational or knowledge facets. The Confidence subscale, although positively correlated with well-being at the zero-order level (*r* = 0.19), had a coefficient statistically indistinguishable from zero in the simultaneous model (β = −0.04, *p* = 0.472); this near-null value does not support a substantive suppression interpretation. Collinearity among the five subscales was low (all VIFs ≤ 1.94). The Physical subscale’s zero-order correlation with well-being (*r* = 0.45) equaled that of the total scale, suggesting that perceived physical competence accounts for much of the total-score association. Because the five-factor measurement model fit the present data only marginally (Section “3.1 Descriptive statistics”), and the scale’s developers intend a single total score, these subdimension results are interpreted as hypothesis-generating (indicating where future latent-variable work might focus) rather than as a definitive localization of the effect.

**TABLE 6:** Zero-order correlations and simultaneous standardized coefficients for the five PLAS subdimensions predicting mental well-being (*N* = 375).

Subdimension	r (FDR q)	β	95% CI	*p*
Motivation	0.34 ( < 0.001)	0.02	[−0.10, 0.14]	0.765
Social	0.34 ( < 0.001)	0.24	[0.14, 0.33]	< 0.001
Physical (coordination/ strength)	0.45 ( < 0.001)	0.40	[0.28, 0.51]	< 0.001
Knowledge	0.17 ( < 0.001)	0.06	[−0.03, 0.15]	0.181
Confidence	0.19 ( < 0.001)	−0.04	[−0.15, 0.07]	0.472

r = zero-order Pearson correlation with mental well-being; q = Benjamini-Hochberg false-discovery-rate-adjusted *p*-value; β = standardized coefficient from the simultaneous regression.

### 3.8 Common-method bias

Because all measures were self-reported from a single source, Harman’s single-factor test was conducted on the 37 PLAS and WEMWBS items. The unrotated solution returned seven factors with eigenvalues exceeding 1.0, and the first (largest) factor accounted for 29.2% of the total variance, well below the 50% threshold conventionally taken to indicate a serious common-method problem (Podsakoff et al., 2003). This result provides no positive evidence of a dominant method factor. Harman’s single-factor test is, however, widely regarded as insensitive: it cannot partition method from trait variance, and a first factor below 50% does not rule out common-method bias (Podsakoff et al., 2003). The present finding should therefore be read as a minimal screening check rather than as confirmation that common-method variance is absent, and the single-source, self-report design is noted among the study’s limitations (Section “4.1 Limitations and future directions”).

## 4 Discussion

This study examined the association between physical literacy and mental well-being in Turkish adults and tested whether the two constructs varied with sex, education, athletic background, regular exercise, and habitual activity level. Three findings stand out. First, physical literacy was moderately and positively associated with mental well-being. Second, behavioral indicators of active living (an athletic background, regular exercise, and especially habitual activity level) differentiated adults on both constructs, with the largest effects for physical literacy. Third, a clear graded gradient linked activity level to both physical literacy and well-being. Together these results extend an association previously documented largely in children and college students (Britton et al., 2023; Ma et al., 2021; Zhu et al., 2025) to a sample of adults. Whereas the only prior adult study contrasted clinically diagnosed and non-diagnosed groups on physical literacy (Naylor et al., 2026), the present findings establish a graded association with positively framed mental well-being across an adult community sample (albeit one skewed toward active and tertiary-educated participants), a segment that has remained underrepresented in the physical-literacy literature (Boldovskaia et al., 2023; Naylor et al., 2024).

The moderate physical-literacy-well-being correlation (*r* = 0.45) is consistent with the holistic conceptualization of physical literacy as a psychosocial resource rather than a narrowly physical capacity (Whitehead, 2001; Cairney et al., 2019). The affective and cognitive components of the construct (confidence and motivation in movement contexts, and the knowledge to act on them) plausibly support the perceived competence, autonomy, and relatedness that, within self-determination theory, constitute the basic psychological needs underpinning eudaimonic well-being (Ryan and Deci, 2000; Tennant et al., 2007). This interpretation aligns with evidence that resilience transmits part of the physical-literacy-mental-health association (Ma et al., 2021; Zhu et al., 2025) and with the parallel mediating role documented for self-esteem (She et al., 2023; Kim and Ahn, 2021). It is further consistent with physical literacy being a robust correlate of physical activity in adults (Naylor et al., 2025; Tang et al., 2023), which is itself causally implicated in better mental health: an umbrella review of randomized trials found medium-sized reductions in depression, anxiety, and distress, with some evidence of greater benefit at higher activity intensities (Singh et al., 2023; Pearce et al., 2022). The shared variance of roughly 20% indicates a meaningful but partial overlap. Because the two constructs are conceptually proximal (particularly the Physical and motivational facets of physical literacy and the positive-functioning items of the WEMWBS), their empirical distinctness cannot be inferred from the moderate correlation alone. The direct test reported in Section “3.2 Association between physical literacy and mental well-being” (HTMT = 0.52, 95% CI [0.42, 0.63]; two-factor model superior to one-factor, ΔCFI = 0.16) indicated that the constructs are empirically distinguishable, which supports the substantive reading of both the association and the incremental-validity finding. Given the study’s dependence on a recently adapted instrument, independent confirmatory replication of this distinction remains a priority. More broadly, the conceptual boundaries of physical literacy remain actively debated. The construct overlaps with, yet is intended to be distinguishable from, neighboring constructs such as physical (or movement) competence, exercise self-efficacy and movement confidence, and health literacy, and commentators continue to disagree over its operationalization and over the degree to which self-report instruments capture its embodied as well as its cognitive elements (Edwards et al., 2017; Cairney et al., 2019; Young et al., 2020; Boldovskaia et al., 2023). This matters for the present interpretation because the WEMWBS indexes positive psychological functioning whereas several PLAS facets, notably Confidence and Motivation, index movement-specific self-perceptions; part of the observed association may therefore reflect this conceptual adjacency rather than a relation between fully separable constructs. The discriminant-validity evidence reported above mitigates but does not eliminate this concern, and the findings are best read with the construct’s contested boundaries in view.

Beyond the bivariate association, the multivariable analyses clarify how physical literacy relates to well-being. Physical literacy explained an additional 11% of variance after demographic and behavioral covariates were accounted for and was the dominant predictor in the adjusted model, satisfying H4 and indicating that its contribution is not reducible to age, sex, education, or sheer activity volume. This increment should, however, be read as an upper bound: physical literacy and well-being share a self-report method and overlap conceptually, so part of the unique variance may be method- and construct-driven rather than evidence of a fully distinct predictor (Section “3.2 Association between physical literacy and mental well-being”). Notably, the unique contributions of athletic background and habitual activity level to well-being were no longer detectable once physical literacy was included in the model (although regular exercise retained a small but significant coefficient). An exploratory analysis indicated that physical literacy statistically accounted for part of the association between habitual activity level and well-being. However, the construct overlap between the predictor and the candidate mediator limits its interpretation. Within self-determination theory, it is plausibly the perceived competence, confidence, and relatedness cultivated through movement (the psychosocial substance of physical literacy) rather than activity frequency *per se*, that carries the well-being benefit (Ryan and Deci, 2000). Self-determination theory is invoked here as an interpretive frame rather than as a tested model: no measure of basic-need satisfaction was collected, and the exploratory analysis operationalized no SDT construct, so the proposed psychological pathway is hypothesized rather than evidenced by the present data. The subdimension analysis further localizes the effect, implicating the Physical (perceived coordination and strength) and Social facets, but not the motivational or knowledge facets, as the unique correlates of well-being, consistent with embodied-competence and social-connectedness accounts of how movement supports mental health. The non-significant interaction indicates that this association is broadly distributed: physical literacy was associated with well-being to a similar degree across activity levels. These exploratory patterns are consistent with the resilience- and self-esteem-mediated pathways previously reported in younger samples (Ma et al., 2021; She et al., 2023; Zhu et al., 2025); because the design is cross-sectional and the predictor and candidate mediator overlap conceptually, however, the ordering remains a hypothesis to be tested rather than a demonstrated causal chain.

The known-groups differences serve a dual purpose. Substantively, they show that cumulative exposure to movement tracks both physical literacy and well-being: adults with an athletic background, regular exercisers, and the most active participants reported the highest scores on both measures, and the activity-level gradient was ordered. The effect sizes also followed a theoretically coherent ordering, being larger for physical literacy, which is closest to the construct’s behavioral core, than for the more distal well-being outcome. Methodologically, these differences provide convergent, known-groups validity evidence for the Turkish PLAS in everyday use, complementing the structural validation previously reported for the Turkish PLAS and reinforcing confidence that the instrument behaves as expected outside its original validation context (Orhan and Karaçam, 2025). This evidence should, however, be weighed against two caveats regarding the instrument. First, the Turkish adaptation resolved into five factors, with the Coordination and Strength factors of the source scale merging into a single Physical factor (Naylor et al., 2024); formal measurement-invariance testing against the original would be required before scores are treated as cross-culturally comparable. Second, because the adaptation has not yet been independently replicated, the present convergent-validity evidence assumes added weight and further independent validation of the Turkish PLAS is warranted. Because the study depends heavily on this instrument and the present authors are also its adapters, we re-estimated the confirmatory factor model on the present data rather than relying solely on the prior validation; fit was modest, with some indices marginal (Section “3.1 Descriptive statistics”), which further underscores the need for independent confirmatory validation of the Turkish PLAS. Until such independent validation is available, the present findings should be regarded as provisional. The modest fit of the five-factor model carries a specific interpretive consequence. It bears most heavily on the subdimension-level findings, which presuppose that the five factors are well-identified, and it counsels that even the more robust total-score associations be read as estimates derived from an instrument still undergoing validation rather than from a settled measure. Accordingly, the substantive conclusions of this study, including the incremental-validity and exploratory mediation results, inherit the measurement uncertainty of the adapted scale and are provisional to that degree. This dependency and the authors’ dual role as adapters and analysts are foregrounded here and fully disclosed in the conflict-of-interest statement.

The small sex difference favoring men on both constructs, and the absence of an education effect on physical literacy, merit measured interpretation. The male advantage in physical literacy is congruent with generally higher rates of sport participation among men in many settings, with evidence that demographic and athletic factors shape women’s self-perceptions in physical activity (Orhan et al., 2025c) and with recent evidence that sociodemographic characteristics predict physical literacy in older women (Mudrinic et al., 2025; Albéniz-Pérez et al., 2025), but the effect was small and the corresponding well-being difference was statistically marginal once unequal variances were accounted for; it should not be over-read. The weak educational gradient in well-being, favoring postgraduate-educated adults, is consistent with the broader association between socioeconomic resources and mental health, but the small effect and the convenience sample temper any strong claim. The negative age-physical-literacy association, paired with a null age-well-being relationship, suggests that declines in perceived physical competence with age need not translate into poorer mental well-being, an observation worth pursuing in age-stratified designs.

From an applied standpoint, the findings position physical literacy as a candidate leverage point for adult health promotion. Because physical literacy is, in principle, modifiable through education and structured experience (Carl et al., 2022; Liu and Chen, 2021), interventions that build movement confidence, motivation, and knowledge, rather than targeting activity volume alone, may yield benefits that extend to mental well-being. Such an approach is well suited to the community health, workplace wellness, and adult education settings for which the Turkish PLAS was proposed (Orhan and Karaçam, 2025). The graded pattern further suggests that even modest increases in activity engagement among the least active may carry disproportionate well-being returns, given that the largest well-being deficits were observed in the no-activity group.

### 4.1 Limitations and future directions

Several limitations qualify these conclusions. First, the cross-sectional design precludes causal or temporal inference; the data are equally consistent with physical literacy fostering well-being, with well-being enabling engagement that builds physical literacy, or with reciprocal and confounded influences. Longitudinal and experimental designs are needed to adjudicate directionality; multi-wave studies of physical activity and psychological distress in adults provide a feasible template. Second, all measures were self-reported and collected from a single source, raising the possibility of common-method variance. However, Harman’s single-factor test indicated that this is unlikely to be a serious threat; future work should incorporate device-based activity assessment and, where feasible, informant or behavioral indices. Because Harman’s single-factor test is a weak diagnostic that cannot partition method from trait variance, it should not be read as evidence that common-method bias is absent; future designs should adopt stronger ex-ante remedies, such as temporal, proximal, or source separation of the predictor and the outcome, alongside ex-post statistical controls, such as an unmeasured-latent or marker-variable method factor, to estimate and adjust for common-method variance more directly. Relatedly, no attention-check or careless-responding items were administered, and because the questionnaire was distributed through an open online link, a participation-rate denominator is unavailable; both constrain the precision of the estimates. Third, the convenience sample was skewed toward men and physically active, highly educated participants, which constrains the external validity of the estimates; probability-based sampling with more balanced sex and activity-level strata would strengthen generalizability to the broader Turkish adult population. In particular, the associations reported here may differ in, and should not be assumed to extend to, less physically active adults, older adults, individuals living with chronic physical or mental-health conditions, and socioeconomically disadvantaged groups, none of which were well represented in this engaged, tertiary-educated sample; whether physical literacy relates to mental well-being to a comparable degree in these populations is an open question that purposive or stratified sampling should address. Fourth, the habitual-activity and sex analyses showed heterogeneity of variance, so the corresponding omnibus tests were confirmed with Welch’s F and Games-Howell or Welch-corrected comparisons; given the magnitude of the activity-level effects, substantive conclusions are unlikely to change. A further measurement limitation is that habitual physical activity, athletic background, and regular exercise were each assessed using single self-report items rather than a validated activity questionnaire (Section “2.2 Measures”); these items capture only activity frequency (days per week), not intensity, duration, or volume. Because this coarse exposure is the backbone of the graded contrasts (H2 and H3) and of the exploratory mediation, the strength of those specific inferences is correspondingly constrained: they should be read as provisional throughout, pending replication with a validated activity measure. Fifth, while the present analyses modeled the five PLAS subdimensions and identified the Physical and Social facets as the unique correlates of well-being, the scale’s developers state that the PLAS is intended to yield a single total score rather than independently interpreted subscale scores; the subdimension analysis should therefore be read as exploratory. A related limitation is that physical literacy and mental well-being are conceptually proximal. However, the discriminant-validity tests reported here indicated that the constructs are empirically distinguishable (HTMT = 0.52); the moderate correlation and the dominance of the Physical subdimension mean that some shared-construct variance cannot be excluded. Latent-variable models that separate measurement error from true-score overlap, ideally within a longitudinal mediation design that also tests resilience or self-efficacy as additional mediators, would provide a more stringent test of the proposed pathway. Finally, the heterogeneous response metrics across PLAS subscales argue for reporting both standardized and raw internal-consistency estimates, which we have done.

## 5 Conclusion

In a sample of Turkish adults, higher physical literacy co-occurred with better mental well-being, and both constructs were patterned by active living, increasing in an ordered gradient across activity-frequency categories. The association and the known-groups differences extend the physical-literacy-well-being link to an underrepresented (though here non-representative) adult sample and provide convergent validity evidence for the Turkish PLAS in applied use. While the cross-sectional, self-report design precludes causal claims, the results identify physical literacy as a theoretically grounded and potentially modifiable target for adult mental-health promotion, and they motivate longitudinal and intervention research, ideally with device-based activity measurement and latent-variable, subdimension-level modeling, to test whether building physical literacy can enhance mental well-being across the adult life course.

## Data Availability

The datasets presented in this study can be found in online repositories. The names of the repository/repositories and accession number(s) can be found in the article/Supplementary material.
