# Supplementary material for: Physical literacy and mental well-being in adults: a cross-sectional examination of their association and links with physical activity engagement
Source: Front Psychol. 2026 Jul 17;17:1908692. doi: 10.3389/fpsyg.2026.1908692 (PMC13423689; doi:10.3389/fpsyg.2026.1908692)
Supplement: Supplementary file 1 [file Supplementary_file_1.docx]

**Supplementary Materials**

*Physical Literacy and Mental Well-Being in Adults: A Cross-Sectional Examination of Their Association and Links with Physical Activity Engagement*

**Status:** All values below were computed from the study dataset (N = 375; complete-case N = 373 for the confirmatory factor models, owing to two missing responses on one Social item, 0.01% of cells). Section numbering (S1-S6) matches the forward references inserted into the revised manuscript. The negatively worded item “I am not physically strong” was reverse-scored on its 1-8 metric before any PLAS score was computed.

**Software:** Descriptives, correlations, and hierarchical regression were computed in Python (pandas, statsmodels) and reproduced the main-text estimates exactly (e.g., Step 3 ΔR² = .110, F(1, 366) = 52.82; βPL = .410; max VIF = 1.89; Durbin-Watson = 1.98). Confirmatory factor models (S2, S3) and the HTMT ratio were estimated by maximum likelihood with the semopy package; indices estimated in AMOS or lavaan may differ marginally.

**Data-integrity check (reviewer Minor 2):** The identical counts for men (n = 242) and regular exercisers (n = 242) were verified against the raw data to be coincidental, not a duplication error: the two subgroups overlap in only 158 cases.

## Contents

S1. Sensitivity analysis: developer-specified (mean-based) PLAS scoring

S2. Discriminant validity: one- vs two-factor CFA and HTMT

S3. Confirmatory factor analysis of the five-factor Turkish PLAS in the present sample

S4. Full hierarchical regression coefficients (all predictors)

S5. STROBE checklist for cross-sectional studies

S6. Software and reproducibility

# S1. Sensitivity Analysis: Developer-Specified (Mean-Based) PLAS Scoring

The primary analyses used a summed-item PLAS total. Because the five subscales differ in response range and item count, summing raw items weights the wider-range subscales (Confidence, maximum 50; Physical, maximum 48) more heavily than the narrower ones (Knowledge, maximum 15). The developers instead specify a total computed as the sum of subscale means (an equal-weighted composite, PLAS_dev; observed M = 24.52, SD = 4.46, range 6.17-35.00). The bivariate correlation and the Step 3 incremental regression were re-estimated with this composite.

**Table S1.** *Primary associations under the two PLAS scoring methods (N = 375).*

| **Statistic** | **Summed-item (reported)** | **Mean-based (developer)** |
| --- | --- | --- |
| r (physical literacy, well-being) | .45 | .45 |
| ΔR² for physical literacy (Step 3) | .11 | .11 |
| β for physical literacy (final model) | .41 | .40 |
| 95% CI for β | [.30, .52] | [.29, .51] |
| p for physical literacy | < .001 | < .001 |

Result: The findings are robust to the scoring choice. Under the developer-specified mean-based composite, the zero-order correlation (r = .446), the incremental variance attributable to physical literacy (ΔR² = .110, p < .001), and the standardised coefficient (β = .399) are materially identical to the summed-item values; direction, significance, and magnitude are unchanged.

**SPSS syntax.**

* Reverse-score the negatively worded Physical item (8-point scale).

RECODE P_neg (1=8)(2=7)(3=6)(4=5)(5=4)(6=3)(7=2)(8=1) INTO P_neg_r.

COMPUTE Motivation_m = MEAN(M1,M2,M3,M4,M5).

COMPUTE Social_m = MEAN(S1,S2,S3,S4).

COMPUTE Confidence_m = MEAN(C1,C2,C3,C4,C5).

COMPUTE Physical_m = MEAN(P1,P_neg_r,P3,P4,P5,P6).

COMPUTE Knowledge_m = MEAN(K1,K2,K3).

COMPUTE PLAS_dev = Motivation_m + Social_m + Confidence_m + Physical_m + Knowledge_m.

EXECUTE.

CORRELATIONS VARIABLES = PLAS_dev WEMWBS_total.

REGRESSION /STATISTICS COEFF CI(95) R ANOVA CHANGE

/DEPENDENT WEMWBS_total

/METHOD = ENTER Age Sex_men Edu_UG Edu_PG

/METHOD = ENTER Athletic Regular Activity

/METHOD = ENTER PLAS_dev.

# S2. Discriminant Validity: One- vs Two-Factor CFA and HTMT

To test whether physical literacy is empirically distinct from mental well-being, a two-factor measurement model (physical literacy indexed by its 23 items as a single latent construct; well-being indexed by its 14 items) was compared with a one-factor model in which all 37 indicators load on a single latent variable. The heterotrait-monotrait (HTMT) ratio, which is robust to the limited absolute fit of single-construct measurement models, is taken as the primary discriminant criterion (threshold < .85).

**Table S2.** *Discriminant validity of physical literacy and mental well-being (complete-case N = 373 for the factor models).*

| **Discriminant-validity test** | **Result** | **Criterion** |
| --- | --- | --- |
| Two-factor vs. one-factor model, χ² difference, Δχ²(1) | 1241.3, p < .001 | Two-factor model superior |
| ΔCFI (two-factor - one-factor) | .16 | Δ > .01 favours two factors |
| HTMT ratio (PL–WB) | .52, 95% CI [.42, .63] | < .85 |
| Latent factor correlation (PL–WB) | .52 | CI excludes 1.0 |

HTMT (physical literacy - well-being) = .52, 95% CI [.42, .63] (discriminant validity supported; threshold < .85).

Discriminant validity is supported. The two-factor model fits significantly and substantially better than the one-factor model, Δχ²(1) = 1241.3, p < .001, ΔCFI = .16, and the HTMT ratio (.52, 95% CI [.42, .63]) is well below the .85 threshold, with the interval excluding 1.0; the estimated latent correlation between the constructs was .52, converging with the HTMT. Together these indicate that physical literacy and mental well-being are empirically distinguishable. Because physical literacy is multidimensional, the single-PL-factor measurement models show limited absolute fit; the comparative evidence and the HTMT, not absolute fit, are therefore the relevant discriminant indices here, consistent with Section 3.2 of the main text.

**R syntax (lavaan / semTools).**

library(lavaan); library(semTools)

two.factor <- '

PL =~ M1+M2+M3+M4+M5+S1+S2+S3+S4+C1+C2+C3+C4+C5+P1+P_neg_r+P3+P4+P5+P6+K1+K2+K3

WB =~ w1+w2+w3+w4+w5+w6+w7+w8+w9+w10+w11+w12+w13+w14

'

one.factor <- '

G =~ M1+M2+M3+M4+M5+S1+S2+S3+S4+C1+C2+C3+C4+C5+P1+P_neg_r+P3+P4+P5+P6+K1+K2+K3 +

w1+w2+w3+w4+w5+w6+w7+w8+w9+w10+w11+w12+w13+w14

'

fit2 <- cfa(two.factor, data = dat, estimator = 'MLR', std.lv = TRUE)

fit1 <- cfa(one.factor, data = dat, estimator = 'MLR', std.lv = TRUE)

fitMeasures(fit2, c('chisq','df','cfi','rmsea','srmr'))

anova(fit1, fit2)

htmt(two.factor, data = dat) # Henseler et al. (2015)

# S3. Confirmatory Factor Analysis of the Five-Factor Turkish PLAS in the Present Sample

Because the study depends heavily on the Turkish PLAS and the present authors are also its adapters, the five-factor structure (Motivation, Social, Confidence, Physical, Knowledge) was re-estimated in the current sample rather than relying solely on the original validation.

**Table S3.** *Fit indices for the five-factor PLAS model (present sample, complete-case N = 373).*

| **Index** | **Present sample** | **Threshold** | **Original validation*** |
| --- | --- | --- | --- |
| χ² (df) | 829.72 (220) | - | - |
| χ² / df | 3.77 | < 3 | 2.06 |
| CFI | .864 | ≥ .90 | .90 |
| TLI | .843 | ≥ .90 | - |
| RMSEA | .086 | ≤ .08 | .06 |
| SRMR | .078 | ≤ .08 | .07 |

*Note.* *Original Turkish validation (Orhan & Karaçam, 2025), reported with AMOS: χ²/df = 2.06, CFI = .90, IFI = .90, RMSEA = .06, SRMR = .07.

The five-factor structure is recovered in the present sample, but with more modest fit than in the original validation: CFI = .864 and TLI = .843 fall just below the conventional .90 criterion, while RMSEA = .086 and SRMR = .078 are near their upper acceptable bounds. The structure is therefore broadly supported but not strongly confirmed, which reinforces the manuscript’s caveats regarding cross-cultural comparability and the need for formal measurement-invariance testing. (ML estimation in semopy; AMOS/lavaan with robust estimation may yield slightly higher incremental indices.)

**R syntax (lavaan).**

library(lavaan)

plas.5f <- '

Motivation =~ M1 + M2 + M3 + M4 + M5

Social =~ S1 + S2 + S3 + S4

Confidence =~ C1 + C2 + C3 + C4 + C5

Physical =~ P1 + P_neg_r + P3 + P4 + P5 + P6

Knowledge =~ K1 + K2 + K3

'

fit <- cfa(plas.5f, data = dat, estimator = 'MLR', std.lv = TRUE)

summary(fit, fit.measures = TRUE, standardized = TRUE)

fitMeasures(fit, c('chisq','df','cfi','tli','rmsea','srmr'))

# S4. Full Hierarchical Regression Coefficients

Table 5 in the main text omitted the education dummies and the athletic-background coefficient for brevity. The complete final-model standardised coefficients are reported here; all reproduce the main-text model exactly (R² = .24, adjusted R² = .22).

**Table S4.** *Complete final-model standardised coefficients predicting mental well-being (N = 375).*

| **Predictor (final model)** | **β** | **95% CI** | **p** |
| --- | --- | --- | --- |
| Age | .13 | [.02, .25] | .022 |
| Sex (men) | .03 | [-.07, .13] | .564 |
| Education: undergraduate vs. high school | -.03 | [-.14, .09] | .670 |
| Education: postgraduate vs. high school | .08 | [-.04, .21] | .173 |
| Athletic background (yes) | .00 | [-.10, .10] | .953 |
| Regular exercise (yes) | .13 | [.01, .25] | .028 |
| Habitual activity level | -.01 | [-.13, .11] | .850 |
| Physical literacy | .41 | [.30, .52] | < .001 |

*Note.* Sex coded 0 = women, 1 = men. Education entered as two dummy codes with high school as the reference. Model R² = .24, adjusted R² = .22, F(8, 366) = 14.55, p < .001. The newly reported rows (education dummies, athletic background) confirm the main-text statement that these predictors were not uniquely significant (all p ≥ .17); only regular exercise and physical literacy retained significant unique coefficients.

The complete table confirms the corrected Results narrative: among the behavioural indicators, only regular exercise (β = .13, p = .028) retained a unique association with well-being once physical literacy entered; athletic background (β = .00) and habitual activity level (β = -.01) did not.

# S5. STROBE Checklist for Cross-Sectional Studies

Completed STROBE checklist (von Elm et al., 2007). The “Location” column gives the manuscript section where each item is addressed; recommendations are paraphrased.

| **Item** | **Topic** | **Recommendation (paraphrased)** | **Location** |
| --- | --- | --- | --- |
| 1 | **Title & abstract** | Indicate design in title/abstract; give a balanced, informative summary. | Title; Abstract |
| 2 | **Background/rationale** | Explain the scientific background and rationale. | 1 |
| 3 | **Objectives** | State objectives and pre-specified hypotheses. | 1 (aims; H1-H4) |
| 4 | **Study design** | Present key design elements early. | Abstract; 2.1 |
| 5 | **Setting** | Describe setting, locations, and relevant dates. | 2.1 |
| 6 | **Participants** | Give eligibility criteria and sources/methods of selection. | 2.1 |
| 7 | **Variables** | Define outcomes, exposures, predictors, confounders. | 2.2 |
| 8 | **Data sources/measurement** | Give data sources and assessment methods for each variable. | 2.2 |
| 9 | **Bias** | Describe efforts to address sources of bias. | 2.3; 3.8; 4.1 |
| 10 | **Study size** | Explain how the study size was arrived at. | 2.1 |
| 11 | **Quantitative variables** | Explain handling of quantitative variables and groupings. | 2.2; 2.3 |
| 12 | **Statistical methods** | (a-e) Methods, subgroups, missing data, sampling, sensitivity analyses. | 2.3; S1-S2 |
| 13 | **Participants (results)** | Numbers at each stage; non-participation; consider flow diagram. | 2.1; 3.1 |
| 14 | **Descriptive data** | Participant characteristics; missing data per variable. | 3.1 (Table 1) |
| 15 | **Outcome data** | Report numbers of outcome events or summary measures. | 3.1 (Table 1) |
| 16 | **Main results** | Unadjusted and adjusted estimates with precision (95% CI). | 3.2-3.7 (Tables 2-6) |
| 17 | **Other analyses** | Report subgroup, interaction, and sensitivity analyses. | 3.6-3.8; S1-S3 |
| 18 | **Key results** | Summarise key results with reference to objectives. | 4 |
| 19 | **Limitations** | Discuss limitations and potential bias. | 4.1 |
| 20 | **Interpretation** | Cautious overall interpretation. | 4 |
| 21 | **Generalisability** | Discuss external validity. | 4.1 |
| 22 | **Funding** | Give the source of funding and the funders’ role. | Declarations |

*Note.* STROBE = Strengthening the Reporting of Observational Studies in Epidemiology. Recommendations paraphrased from von Elm et al. (2007), Lancet, 370, 1453-1457. Update section references if the manuscript is re-paginated.

# S6. Software and Reproducibility

Primary analyses (descriptives, reliabilities, correlations, t-tests, ANOVAs, hierarchical regression, PROCESS mediation) were conducted in IBM SPSS Statistics (v.25) with the PROCESS macro (v4.2); these were independently reproduced in Python (pandas, statsmodels), matching the reported estimates. The confirmatory factor models (S2, S3) and the HTMT ratio were estimated by maximum likelihood (semopy in Python; equivalently lavaan in R). Bootstrap analyses used 5,000 resamples with a fixed seed (31216). Internal-consistency estimates recomputed here matched the main text (Social α = .81, PLAS total α = .89, WEMWBS α = .92).
